# HDAC3 inhibition prevents blood-brain barrier permeability through Nrf2 activation in type 2 diabetes male mice

**DOI:** 10.1186/s12974-019-1495-3

**Published:** 2019-05-17

**Authors:** Qiuchen Zhao, Fang Zhang, Zhanyang Yu, Shuzhen Guo, Ning Liu, Yinghua Jiang, Eng H. Lo, Yun Xu, Xiaoying Wang

**Affiliations:** 1 Department of Neurology, Affiliated Drum Tower Hospital, Medical School of Nanjing University, 321 Zhongshan Rd, Nanjing, 210008 Jiangsu China; 2 Department of Neurology, Tianjin Neurological Institute, Tianjin Medical University General Hospital, Tianjin, 300052 China; 3 Neuroprotection Research Laboratory, Departments of Radiology and Neurology, Massachusetts General Hospital and Harvard Medical School, 149 13th Street, Room 2401, Charlestown, Boston, MA 02129 USA; 4 The Third Affiliated Hospital of Zhengzhou University, Zhengzhou, 450052 Henan China

**Keywords:** Neuroinflammation, Histone deacetylase 3 (HDAC3), Diabetes, Blood-brain barrier (BBB), Hyperglycemia, Interleukin 1 beta (IL1β), Nuclear factor-E2-related factor 2 (Nrf2)

## Abstract

**Background:**

Type 2 diabetes mellitus (T2DM) is a chronic metabolic dysfunction characterized by progressive insulin resistance and hyperglycaemia. Increased blood-brain barrier (BBB) permeability is a critical neurovascular complication of T2DM that adversely affects the central nervous system homeostasis and function. Histone deacetylase 3 (HDAC3) has been reported to be elevated in T2DM animals and may promote neuroinflammation; however, its involvement in the BBB permeability of T2DM has not been investigated. In this study, we tested our hypothesis that HDAC3 expression and activity are increased in the T2DM mouse brain. Inhibition of HDAC3 may ameliorate T2DM-induced BBB permeability through Nrf2 activation.

**Methods:**

T2DM (db/db, leptin receptor-deficient), genetic non-hyperglycemic control (db/+), and wild-type male mice at the age of 16 weeks were used in this study. HDAC3 expression and activity, Nrf2 activation, and BBB permeability and junction protein expression were examined. The effects of HDAC3 activity on BBB permeability were tested using highly selective HDAC3 inhibitor RGFP966. In primary cultured human brain microvascular endothelial cells (HBMEC), hyperglycemia (25 mM glucose) plus interleukin 1 beta (20 ng/ml) (HG-IL1β) served as T2DM insult in vitro. The effects of HDAC3 on transendothelial permeability were investigated by FITC-Dextran leakage and trans-endothelial electrical resistance, and the underlying molecular mechanisms were investigated using Western blot, q-PCR, co-immunoprecipitation, and immunocytochemistry for junction protein expression, miR-200a/Keap1/Nrf2 pathway regulation.

**Results:**

HDAC3 expression and activity were significantly increased in the hippocampus and cortex of db/db mice. Specific HDAC3 inhibition significantly ameliorated BBB permeability and junction protein downregulation in db/db mice. In cultured HBMEC, HG-IL1β insult significantly increased transendothelial permeability and reduced junction protein expression. HDAC3 inhibition significantly attenuated the transendothelial permeability and junction protein downregulation. Moreover, we demonstrated the underlying mechanism was at least in part attributed by HDAC3 inhibition-mediated miR-200a/Keap1/Nrf2 signaling pathway and downstream targeting junction protein expression in T2DM db/db mice.

**Conclusions:**

Our experimental results show that HDAC3 might be a new therapeutic target for BBB damage in T2DM.

**Electronic supplementary material:**

The online version of this article (10.1186/s12974-019-1495-3) contains supplementary material, which is available to authorized users.

## Introduction

T2DM has been regarded as a risk factor and important cause of multiple neurological disorders including dementia, cerebrovascular disease, and anxiety/depression, therefore has been a focus of study on metabolism dysfunction-induced neurological diseases [1]. The blood-brain barrier (BBB) is a protective mechanism that maintains cerebral homeostasis and provides the central nervous system with unique protection against foreign matters [2]. BBB is located at the level of the capillaries between the blood and cerebral tissue and is characterized by the presence of tight intracellular junctions and polarized expression of many transport systems [3]. BBB damage is a critical neurovascular complication of T2DM that adversely affects the central nervous system (CNS) function. In vitro and in vivo studies have shown that diabetes could lead to the impairment of BBB integrity and subsequent BBB permeability increase [4, 5]. Hence, the development of therapeutics targeting BBB damage in diabetes has been considered clinically significant [6].

Histone acetylation and deacetylation are two major epigenetic mechanisms in the regulation of gene transcription. In general, acetylation of histone by histone acetyltransferases (HATs) is thought to weaken the interaction of histone with DNA, thus providing greater accessibility of regulatory elements to DNA and promote gene transcription. On the other hand, histone deacetylases (HDACs) catalyzes the deacetylation of histone proteins, hence inhibiting gene transcription [7, 8].

HDAC3 is the most highly expressed class I HDAC in the brain, predominantly expressed in the hippocampus, cortex, and cerebellum [9]. HDAC3 plays multiple roles in a variety of pathophysiological conditions including cancer and neurodegenerative diseases. Experimental studies show that HDAC3 inhibitor suppresses cell apoptosis induced by inflammatory cytokines or glucolipotoxic stress and increase insulin release in vitro [10]. Moreover, HDAC3 inhibition has also been shown to improve plasma glucose levels and pancreatic β cell function in a rat model of type 2 diabetes [11]. Interestingly, HDAC3 activity was significantly increased in the peripheral blood mononuclear cells of patients with T2DM compared to control subjects, and its activity/mRNA levels positively correlated with proinflammation, poor glycemic control, and insulin resistance [12]. These emerging experimental findings imply that HDAC3 might play important roles in diabetic pathophysiology and complications [13].

Nuclear factor-E2-related factor 2 (Nrf2) is a redox-sensitive master regulator of the expression of antioxidant proteins that protect against oxidative damage triggered by injury and inflammation [14]. Multiple studies have established the protective role of Nrf2 against BBB damage in various CNS pathologies [15, 16]. It has been reported HDAC3 inhibition regulates Keap1/Nrf2 balance through modulating the expression of miR-200a, which binds to the 3′-terminal region of the Keap1 mRNA to downregulate its translation [17], and the reduced Keap1 level leads to an increase in Nrf2 nuclear translocation, which subsequently increases the transcription of antioxidant and anti-inflammatory genes [18]. Very interestingly, our recent experimental study showed HDAC3 inhibition prevented oxygen-glucose deprivation/reoxygenation (OGD/R)-induced transendothelail cell permeability increase in cultured human brain microvascular endothelial cells (HBMEC), suggesting an important role of HDAC3 in BBB permeability modulation [19]. In diabetic subjects, Nrf2 dysregulation may mediate oxidative/inflammatory stress-induced neurovascular dysfunction and BBB disruption [20]. Our recent study has demonstrated that BBB leakage protection by recombinant FGF21 treatment was mainly contributed through Nrf2 upregulation in type 2 diabetic mice [21].

In this study, we tested our hypothesis that HDAC3-specific inhibitor (RGFP966) may reduce T2DM-induced BBB permeability via Nrf2 activation. Results from this study showed that HDAC3 activity and expression in the hippocampus and cortex were increased in db/db mice. HDAC3 inhibition protected against diabetes-induced BBB permeability. In cultured HBMEC, we further revealed that HDAC3 inhibition repressed Keap1 through miR-200a upregulation, thereby reducing Keap1-Nrf2 interaction and promoting Nrf2 activation, leading to the protective effects against T2DM-induced BBB permeability.

## Material and methods

All animal experiments were performed following the protocols approved by the Massachusetts General Hospital Animal Care and Use Committee in compliance with the National Institutes of Health Guide for the Care and Use of Laboratory Animals. Experimenters were blinded to data acquisition.

### Animals and drug treatments

Male leptin receptor-deficient mice (db/db, stock no: 000642), control mice (db/+, stock no: 000642), and wild-type mice (C57BLKS/J, stock no: 000662) were purchased from The Jackson Laboratory (Jackson Laboratory, Bar Harbor, ME) at ages of 16 weeks. A table listed the animal groups and numbers used in each experimental assessment (Additional file 1). The animals were randomly assigned to vehicle-treated or RGFP966 (Cat Number: S7229; Selleck Chemicals, Houston, TX) treatment groups. RGFP966 (or vehicle) was administered at the dose of 10 mg/kg/day by intraperitoneal injection for 10 days. The dose selection was based on previously established dose regimen of RGFP966 treatment in mice [8].

### Preparation of brain microvascular

Brain microvascular was isolated following an established method as we previously described [22]. Briefly, after cardiac perfusion with phosphate-buffered saline (PBS), the hippocampus and cortex were harvested, then homogenized in cold PBS on ice with Knote Dounce glass tissue grinder (Part 885300-0002; Kimble Chase Life Science, Vineland, NJ) and centrifuged at 4 °C, 500*g* for 5 min. The tissue pellet was suspended with 18% Dextran solution (molecular weight 60–90 kDa; USB Corporation, Cleveland, OH) in PBS and then centrifuged again at 4 °C, 2500*g* for 20 min. The final suspension was filtered through a 40-μm cell strainer to get rid of remaining single cells or small cell clumps. The resulting microvascular on the top of the cell strainer was used directly for RNA extraction. Our previous study has demonstrated these isolated brain microvascular fragments are positive for endothelial CD31 marker immune staining and lack contaminating signals from astrocytic GFAP or smooth muscle/pericyte a-SMA markers [22].

### Mice BBB permeability

The in vivo sodium fluorescein (NaFl)/fluorescein isothiocyanate (FITC)-Dextran permeability assay was performed as we previously described [21]. Briefly, to assess BBB permeability with NaFl, mice were introperitoneally injected with NaFl at the dose of 200 mg/kg at day 10 after RGFP966 treatment. One hour after NaFl injection, mice were sacrificed by transcardial perfusion with saline under isoflurane anesthesia. The hippocampus and cortex were isolated, and NaFl was extracted using 30% trichloroacetic acid. NaFl fluorescence (excitation 440 nm and emission 525 nm) was quantified using fluorescence microplate reader (SpectraMax M5, Molecular Device). To assess BBB permeability with FITC-Dextran (4 kDa or 10 kDa), mice were introperitoneally injected with FITC-Dextran at the dose of 50 mg/kg. One hour after FITC-Dextran injection, mice were sacrificed by transcardial perfusion with saline under isoflurane anesthesia. The hippocampus and cortex were isolated, and FITC-Dextran was extracted using phosphate-buffered saline. FITC-Dextran fluorescence (excitation 490 nm and emission 520 nm) was quantified using fluorescence microplate reader (SpectraMax M5, Molecular Device). Fluorescence values from duplicate wells were fit to a standard curve, averaged, and expressed relative to the total protein amount as determined by the Bradford assay. For the visualization of fluorescence penetration in the brain sections, after transcardial perfusion with saline, the brains were post-fixed in 4% PFA for 1 h. Next, 20 μm sagittal slices were obtained with a Leica freezing microtome and staining with rat anti-CD31 antibody (Cat Number: 550274; BD Bioscience, San Jose, CA). Fluorescent signals were examined using Nikon Eclipse T300 fluorescence microscope.

### Human brain microvascular endothelial cell culture and viability

Primary HBMEC was purchased from Cell Systems Corporation (ACBRI376, Kirkland, WA) and cultured in complete growth media EBM-2 with supplement growth factors (Lonza, Walkersville, MD). The viability of cultured HBMEC was assessed by WST assay in 24-well plates as we previously described [23]. WST-1 reagent (50 μl) was added to each well (containing 450 μl medium) and incubated for 2 h at 37 °C. Absorbance was determined at 490 nm against untreated cells using a microplate reader (SpectraMax M5, Molecular Devices) according to the manufacturer’s instruction.

### FITC-Dextran transendothelial permeability assay

FITC-Dextran transendothelial permeability assay was performed as we previously described [21]. Briefly, primary HBMEC was cultured on the inner surface of collagen-coated Transwell inserts (6.5 mm diameter, 0.4 μm pore size; Corning, NY) in complete EBM-2 containing normal (5 mM) glucose or 25 mM glucose (hyperglycemia). HBMECs were seeded at a density of 2 × 10^4^ cells/well in 200 μl medium onto 24-well Transwell chambers. The cell monolayer normally reaches confluency after 2 days. Then, the hyperglycemia-treated cells were switched to hyperglycemia EBM without FBS or growth factors but with IL-1β (20 ng/ml) for 16 h. For RGFP966 (final concentration 5 μM) or trigonelline (final concentration 1 μM; Cat Number: T5509; Sigma, St. Louis, MO) treated group, these drugs were added to the medium immediately right after IL-1β exposures. The media in both upper and lower chambers were then removed and replaced with fresh media without supplement. Permeability was measured by adding 0.1 mg/ml of FITC-labeled Dextran (Cat Number: 46945, MW, 70,000; Sigma, St. Louis, MO) to the upper chamber, with the lower compartment containing fresh serum-free media. After incubation for 20 min, 100 μl of medium from the lower compartment was taken and measured for fluorescence at excitation 490 nm and emission 520 nm using a microplate reader (SpectraMax M5, Molecular Devices). All independent experiments were performed in triplicate.

### Measurement of trans-endothelial electrical resistance

The trans-endothelial electrical resistance (TEER) was measured as we previously described [23]. The same experimental groups tested for permeability assay were examined for TEER. In brief, the 6 mm Transwell with cultured endothelial cell monolayer was transferred into the EndOhm-6 chamber. Both the media in the chamber and Transwell were replaced with 0.1 M KCl. EndOhm cap was then inserted on the top of the chamber and Transwell connected with the chamber using a connector cable, and resistance was then measured using the EVOM resistance reader (World Precision Instruments, Sarasota, FL). Meanwhile, a new Transwell containing 0.1 M KCl without any cells was used as a blank control.

### Western blots

Since Nrf2/Keap1 pathway is a redox-sensitive master regulator, it has been reported that Nrf2/Keap1-junction protein expression cascade plays an important role in BBB permeability. To draw a potential causality, we thus sampled brain tissues for the assessment of upstream regulators Nrf2/Keap1 at early day 7 and its downstream junction protein expression at late day 10 with/without HDAC3 inhibition, respectively. For brain sample protein extraction, the mouse was cardio-perfused with saline. Then, the hippocampus and cortex protein were extracted using lysis buffer (Cat Number: 9803, Cell Signaling, Danvers, MA). For total protein isolation of cultured HBMEC, the cells were washed twice with ice-cold PBS then lysed with ice-cold lysis buffer. For junction proteins examination, plasma membrane protein was isolated using the Plasma Membrane Protein Extraction Kit (Cat Number: ab65400, Abcam). The proteins were separated on 4–20% Tris-glycine gel, followed by blotting to the PVDF membrane. The membrane was then incubated with primary anti-HDAC3 (1:1000, Cat Number: ab32369, RRID: AB_732780, Abcam), anti-Histone H3 (1:1000, Cat Number: 4499, RRID: AB_10544537, Cell Signaling), anti-GAPDH (1:10,000, Cat Number:5174, RRID: AB_10622025, Cell Signaling), anti-Na,K-ATPase(1:1,000, Cat Number: ab7671, RRID: AB_306023, Abcam), anti-Nrf2 (1:1,000, Cat Number: 12721, RRID: AB_2715528, Cell Signaling), anti-Keap1 (1:1,000, Cat Number: sc-33569, RRID: AB_2280949, Santa Cruz), anti-IL-1β (1:1,000, Cat Number: 31202,Cell Signaling), anti-Claudin-5 (1:1000, Cat Number: ab15106, RRID: AB_301652, Abcam), anti-VE-cadherin (1:1000, Cat Number: ab33168, RRID: AB_870662, Abcam), anti-Occludin (1:1000, Cat Number: 71-1500, RRID: AB_2533977, Invitrogen), and anti-ZO-1 (1:1000, Cat Number: 617300, RRID: AB_2533938, Invitrogen) antibodies at 4 °C overnight. The membrane was incubated with horseradish peroxidase-conjugated secondary antibodies and washed with TBST, and immunolabeling was detected by enhanced chemiluminescence (ECL; GE Healthcare) according to the manufacturer’s instruction. Quantitative densitometry was performed on the protein bands by using ImageJ software.

### RNA purification and quantitative PCR

For HDAC3 mRNA expression, brain tissue samples of the hippocampus and cortex were collected from mice at the age of 16 weeks. For miRNA200a and U6snRNA expression, brain tissue samples were collected after day 7 with/without RGFP966 treatment. For Nrf2-targeted pro-inflammatory cytokines expression, brain tissue samples were collected after day 10 with/without RGFP966 treatment. Following transcardial perfusion with 0.01 M phosphate-buffered saline (pH 7.4), hippocampus and cortex tissues of mice were collected and stored at − 80 °C until processing. Total RNA was extracted and reverse-transcribed using RNeasy Lipid Tissue Mini Kit (Qiagen) and QuantiTect reverse transcription system (Qiagen) according to the manufacturer’s instructions. Real-time polymerase chain reaction (PCR) was performed on an ABI 7500 Fast Real-Time PCR system using TaqMan gene expression assays for HDAC3 (Cat Number: Mm00515916_m1), HO-1 (Cat Number: Mm00516005_m1), CAT (Cat Number: Mm00437992_m1), and housekeeping gene B2M (Cat Number: Mm00437762_m1) (Applied Biosystems, USA). Levels of miRNA200a (Cat Number: 000502) and U6snRNA control RNA (Cat Number: 001973) were measured using the TaqMan microRNA Reverse Transcription Kits (Cat Number:4366596, Thermo Fisher) and ABI 7500 Fast Real-Time PCR system according to the manufacturer’s instructions. Reactions were performed in duplicate according to the manufacturer’s instructions. Relative expression levels were calculated with the 2 − ΔΔCt method.

### HDAC3 activity assay

Briefly, the nuclear protein was extracted from the hippocampus and cortex using the NE-PER™ Nuclear and Cytoplasmic Extraction Reagents (Cat Number: 78835, Thermo Fisher Scientific, Waltham, MA). Nuclear protein concentration was measured and normalized with a selective internal control Histone H3. HDAC3 activity was quantified with HDAC3 Activity Assay Kit (Cat Number: EPI004, Simga, St. Louis, MO) following the manufacturer’s instruction.

### Immunocytochemistry

Immunostaining for junction proteins in HBMEC was performed following a standard method as we previously described [21]. Briefly, cultured HBMEC in 24-well plates were washed with PBS and fixed with 4% paraformaldehyde for 30 min, then washed with PBS containing 0.1% Tween and further incubated with 5% FBS for 1 h. Next, the cells were incubated with primary anti-Claudin-5 (1:1000, Cat Number: ab15106, RRID: AB_301652, Abcam), anti-VE-cadherin (1:1000, Cat Number: ab33168, RRID: AB_870662, Abcam), and anti-ZO-1 (1:1000, Cat Number: 617300, RRID: AB_2533938, Invitrogen) antibodies at 4 °C overnight. After PBS washing, the cells were incubated with fluorescence-conjugated secondary antibodies (Jackson ImmunoResearch), for 1 h at room temperature. Vectashield mounting medium containing DAPI was used to cover the wells. Fluorescent signals were examined using Nikon Eclipse T300 fluorescence microscope.

### Co-immunoprecipitation

Co-immunoprecipitation (Co-IP) was performed following our previously published methods [21]. Proteins were extracted from mice at day 7 or cultured HBMEC, and immunoprecipitation was performed using 2 μg polyclonal antibody against mouse Keap1 (1:1,000, Cat Number: sc-33569, RRID: AB_2280949, Santa Cruz). After 3 hr incubation, protein G sepharose was added and incubated overnight at 4 °C, and then centrifuged for 1 min at 12,000*g*. The precipitates were rinsed with immunoprecipitation buffer (0.5% NP-40, Tris-Cl pH 8.0, 0.15 M NaCl) four times to remove non-specific binding molecules. IgG was used as a negative control for precipitation. The protein levels of Nrf2 and Keap1 in precipitates were then assessed by Western blot.

### Statistical analysis

SPSS for Windows version 17.0 software (SPSS, Inc, Chicago, IL, USA) was used for the data analyses. Results were expressed as mean ± SEM. Abnormal distribution was compared as groups using a Mann-Whitney test. Two-tailed unpaired *t* test was used for the two groups. One-way ANOVA was used for comparison of three or more groups. *P* < 0.05 was considered statistically significant.

## Results

### HDAC3 expression and activity are increased in the brain of db/db mice

**Fig. 1:**
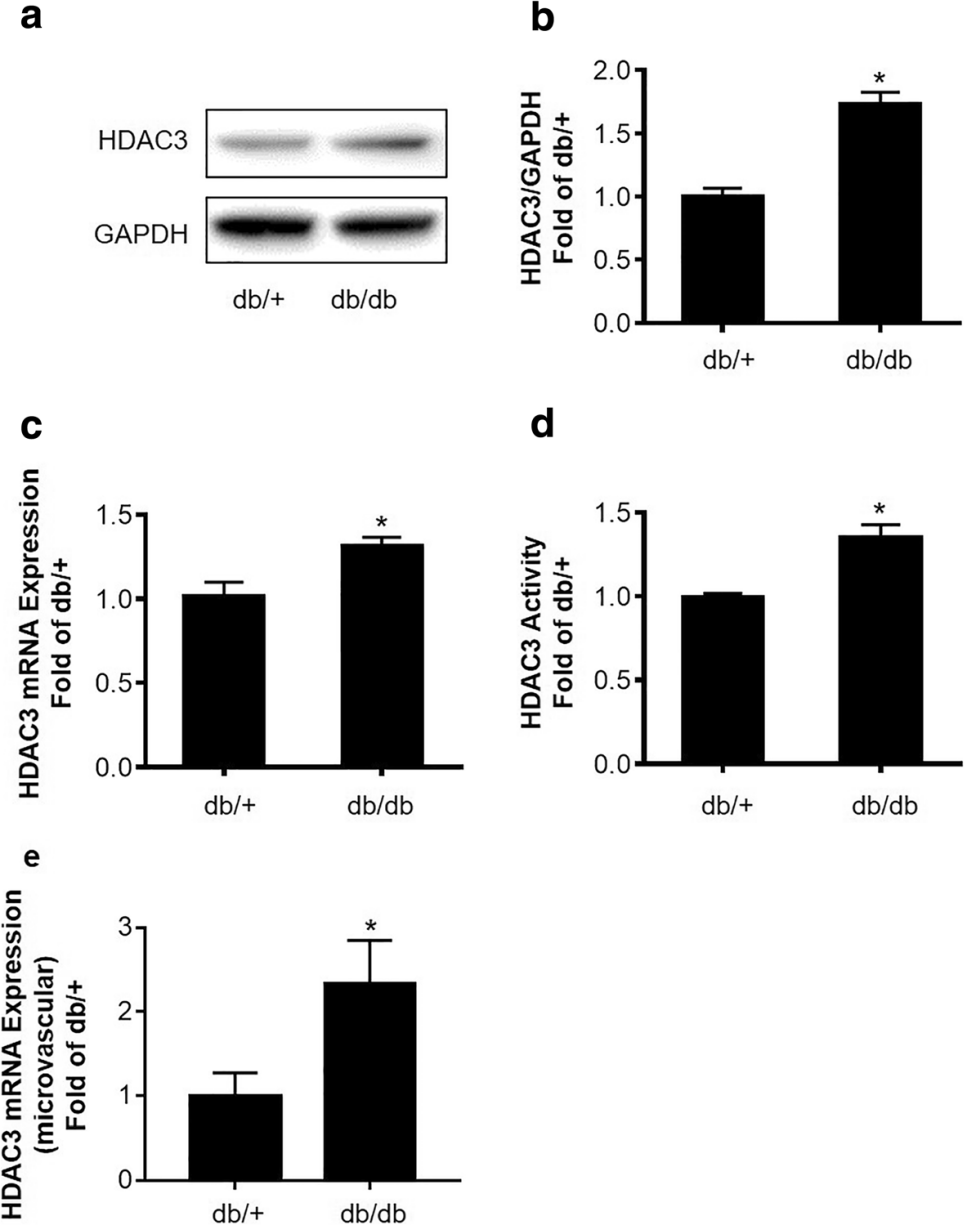
HDAC3 expression and activity are increased in the brain in db/db mice. **a** Representative Western blots gel images of HDAC3 expression in the mouse brain hippocampus and cortex tissues. **b** Quantification of Western blot analysis of HDAC3 protein expression. GAPDH served as an equal loading control. **c** Quantification of HDAC3 mRNA expression examined by real-time quantitative PCR. **d** Quantification of HDAC3 activity in nuclear extraction of brain hippocampus and cortex tissues. **e** Quantification of HDAC3 mRNA expression in brain microvascular examined by real-time quantitative PCR. Data are expressed as mean ± SEM, *P < 0.05 vs. db/+, *n* = 6 mice per group

Since BBB leakage in type 2 diabetes model was reported mainly located in the brain hippocampus [24] and cortex [25], mixed hippocampus and cortex tissue sample was used in this study. HDAC3 mRNA and protein expression levels were significantly increased in the db/db group compared to the db/+ group (Fig. 1a–c). We for the first time also examined the HDAC3 activity between the db/db and db/+ mouse brains. HDAC3 activity was also significantly higher in db/db compared to db/+ mice (Fig. 1d). In addition, we isolated the brain microvascular and measured HDAC3 mRNA expression. Consistent with the results obtained from mixed brain tissue samples of the hippocampus and cortex, HDAC3 mRNA expression in microvascular was significantly elevated in the db/db group (Fig. 1e). Then, we also assessed and compared the baseline levels of HDAC3 expression and activity between db/+ mice and genetic matching wild-type mice (C57BLKS/J); no baseline differences were detected (Additional file 3).

### HDAC3 inhibition reduces diabetes-induced BBB permeability and rescues junction protein expression in db/db mice

**Fig. 2:**
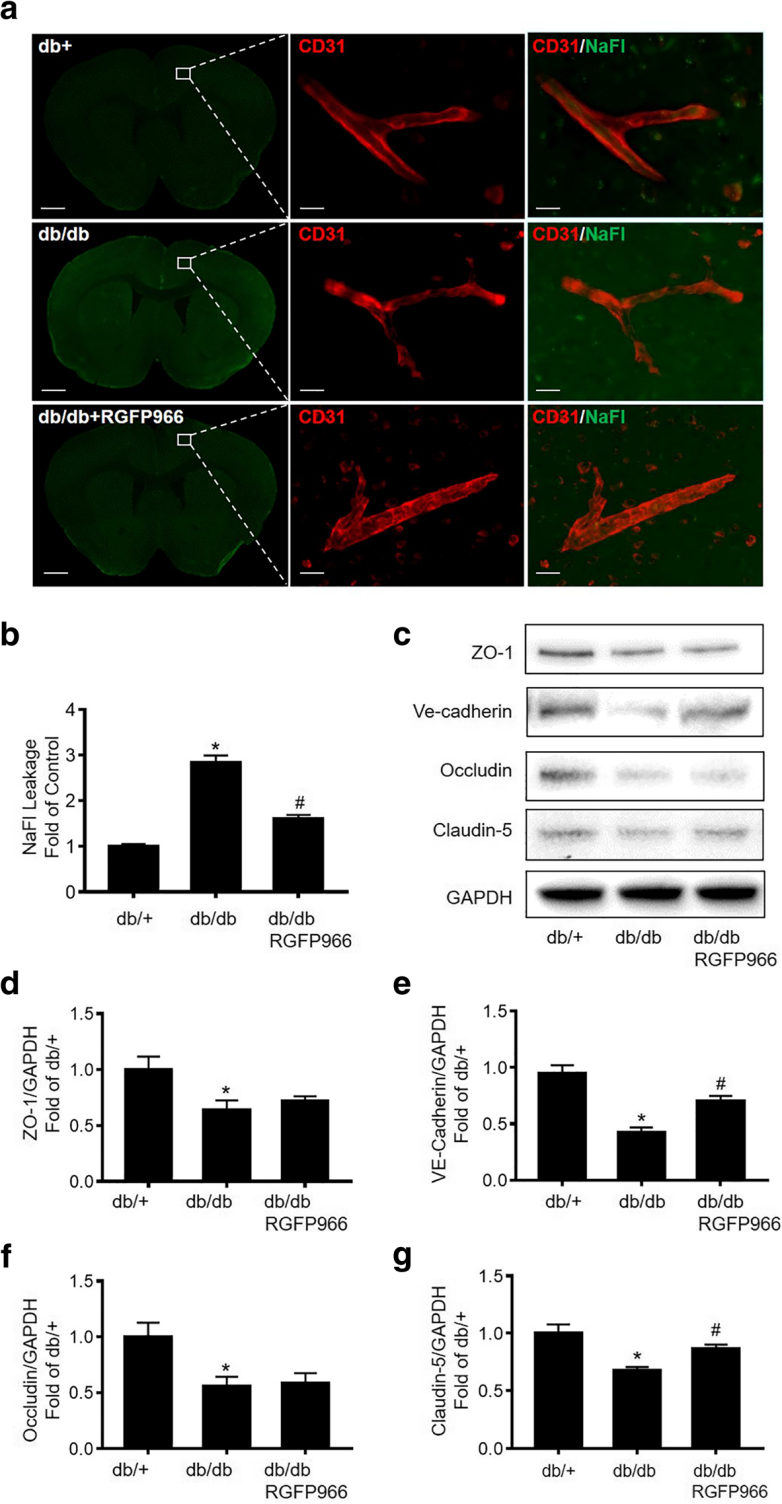
HDAC3 inhibition reduces diabetes-induced BBB permeability and rescues junction protein expression in db/db mice. **a** db/db mice at the age of 16 weeks were treated with RGFP966 for 10 days. Fluorescent images of sodium fluorescein extravasation and CD31 staining. Images show × 4 macro-shots for the whole brain as well as zoomed in × 20 images in representative regions. **b** NaFl leakage was determined by quantifying the relative fluorescent intensity of sodium fluorescein. **c** Representative Western blot gel images of junction proteins ZO-1, VE-cadherin, Occludin, and Claudin-5. **d**–**g** Western blot analysis quantification for protein expression of ZO-1, VE-cadherin, Occludin, and Claudin-5. Data are expressed as mean ± SEM. *P < 0.05 vs. db/+, ^#^*P* < 0.05 vs. db/db, *n* = 5 mice per group

We used fluorescent tracers NaFl (M.W 376), as well as FITC-Dextran (4 kDa and 10 kDa, respectively) to examine BBB permeability. We found that BBB permeability of NaFl was significantly increased in db/db compared to db/+ mice (Fig. 2a, b). However, there were no detectable leakages of bigger tracers FITC-Dextran (4 kDa, and 10 kDa, respectively). Moreover, HDAC3 inhibition by RGFP966 (10 mg/kg/day for 10 days) significantly decreased NaFl permeability compared to the vehicle group (Fig. 2a, b). And RGFP966 treatment for 10 days did not change the blood glucose level or body weight in db/db mice (Additional file 2). Tight junction and adheren junction proteins play important roles in maintaining BBB integrity [26]. We then tested the junction proteins expression by Western blot. We found that ZO-1, VE-Cadherin, Occludin, and Claudin-5 protein levels were significantly decreased in db/db mice compared to db/+ mice, while HDAC3 inhibition significantly rescued the expression of VE-Cadherin and Claudin-5 (Fig. 2c–g).

### HDAC3 inhibition reduces hyperglycemia-IL1β insult-induced permeability of cultured HBMEC monolayer

**Fig. 3:**
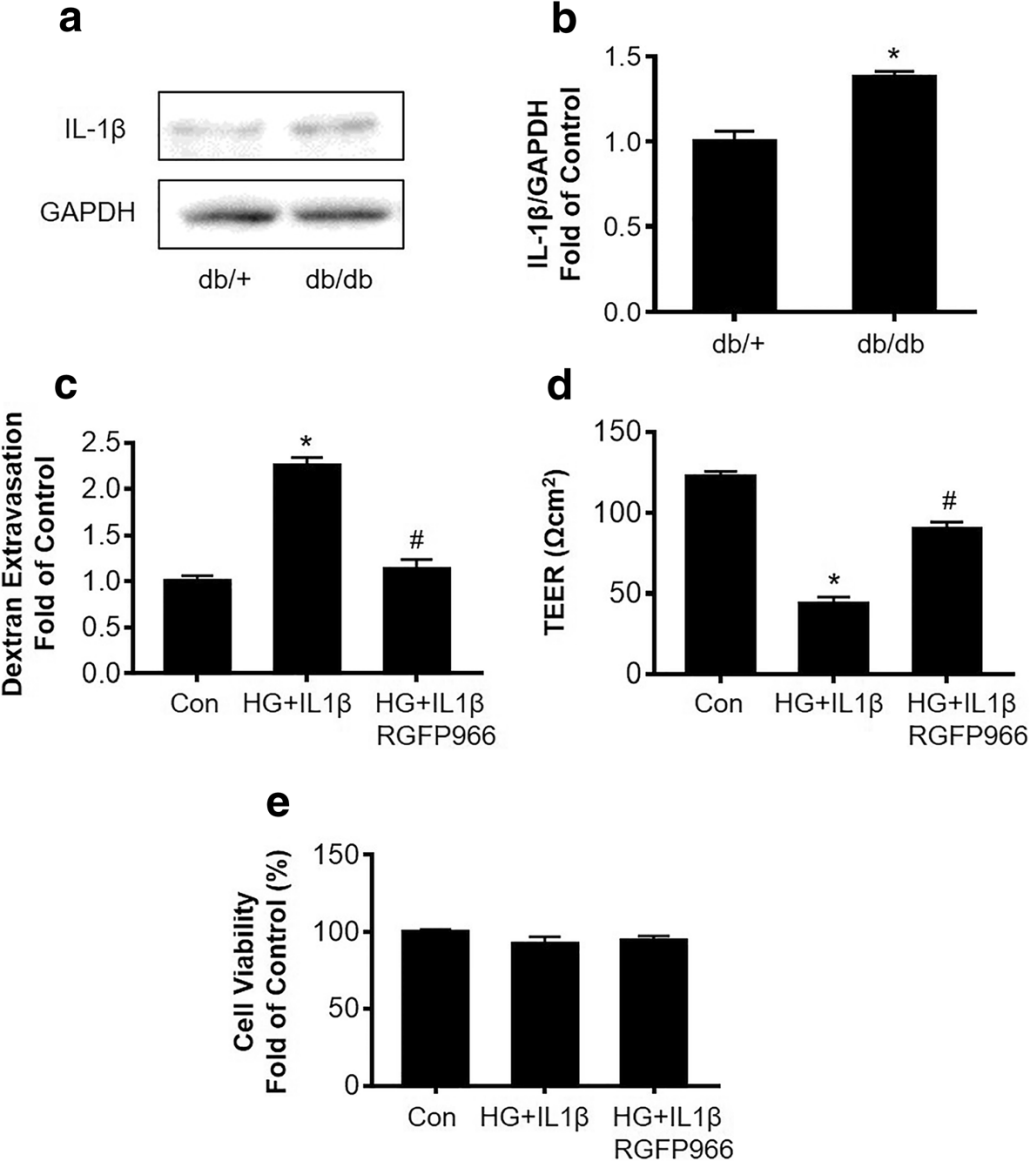
HDAC3 inhibition reduces hyperglycemia-IL1β (HG-IL1β) insult-induced permeability of cultured HBMEC monolayer. **a** Representative Western blot gel images of IL-1β expression in the mouse brain hippocampus and cortex tissues. **b** Quantification of Western blot analysis of IL-1β protein expression. Data are expressed as mean ± SEM. *P < 0.05 vs. db/+, *n* = 4; c. Relative HBMEC monolayer permeability after HG-IL1β insult with/without RGFP966 treatment. **d** TEER measurement of HBMEC monolayers after HG-IL1β insult with/without RGFP966 treatment. **e** Viability of cultured HBMEC after HG-IL1β insult with/without RGFP966 treatment. Data are expressed as mean ± SEM. *P < 0.05 vs. norm, ^#^*P* < 0.05 vs. HG-IL1β, *n* = 6

We found that IL-1β protein level was significantly increased in the db/db mouse brains compared to db/+ mice, suggesting an upregulated inflammation in the db/db mouse brain (Fig. 3a, b). Thus, we used hyperglycemia (25 mM glucose) plus IL-1β (20 ng/ml) (HG-IL1β) in HBMEC monolayer culture as an in vitro model of diabetic BBB insult [21]. HG-IL1β insult significantly increased the permeability of HBMEC monolayer, whereas this increase was significantly reversed by HDAC3 inhibition (Fig. 3c). To further validate the effect of HDAC3 inhibition on endothelial monolayer integrity, we tested the TEER using the EndOhm chamber and EVOM resistance meter. We found that HG-IL1β insult significantly decreased the TEER of cultured HBMEC monolayer compared to normal control, whereas HDAC3 inhibition significantly rescued the TEER decrease compared to HG-IL1β group, further validating the protective effect of HDAC3 inhibition on endothelial monolayer integrity (Fig. 3d). HG-IL1β or HG-IL1β+RGFP966 (5uM) did not significantly change the viability of HBMECs, suggesting that the protective effect of RGFP966 against HG-IL1β-induced endothelial monolayer permeability is not through increasing the cell viability (Fig. 3e).

### HDAC3 inhibition prevents hyperglycemia-IL1β insult-induced tight junction loss in cultured HBMEC monolayer

**Fig. 4:**
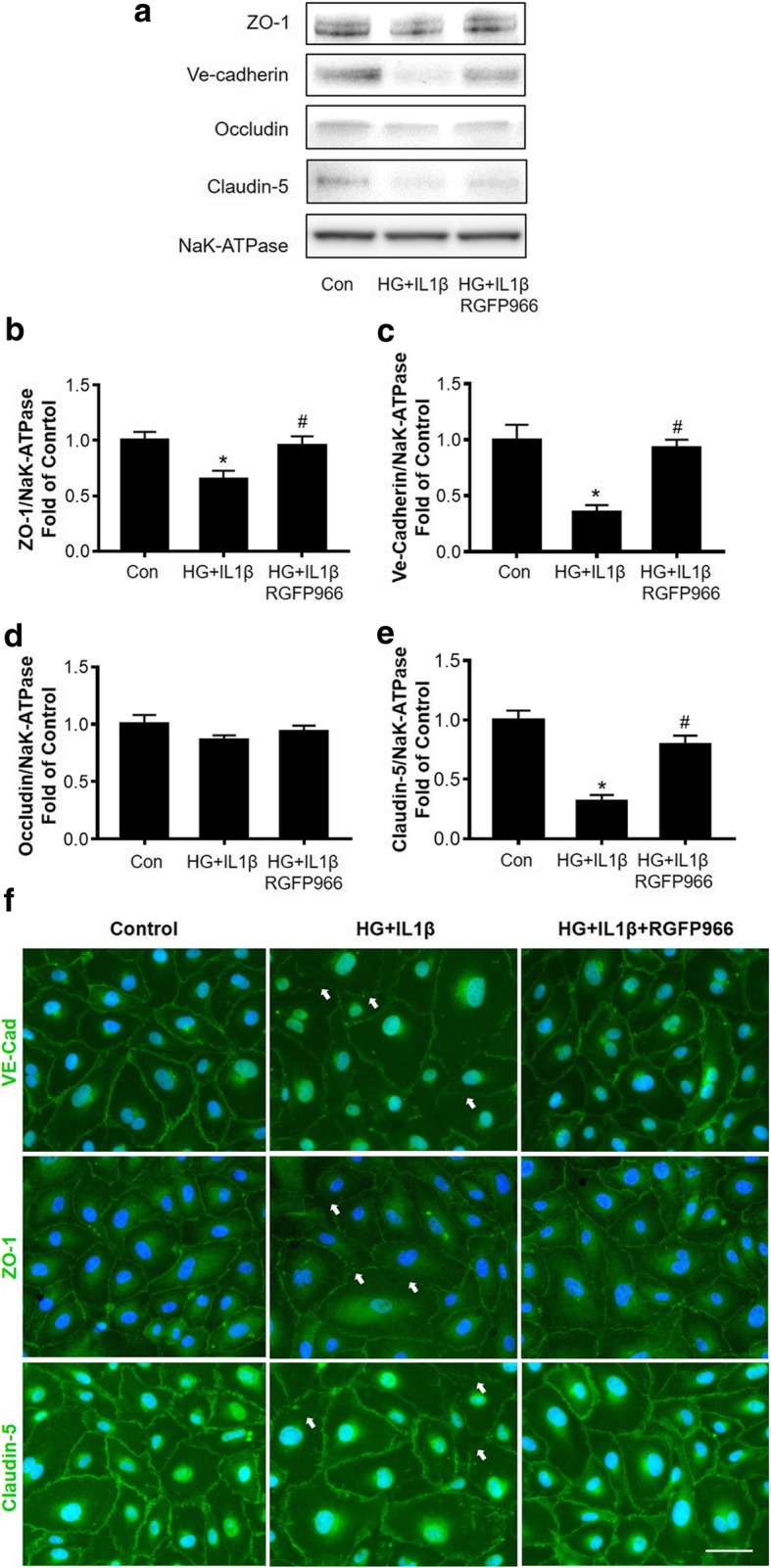
HDAC3 inhibition prevents hyperglycemia-IL1β (HG-IL1β) insult-induced tight junction loss in cultured HBMEC monolayer. **a** Representative Western blot gel images of ZO-1, VE-cadherin, Occludin, and Claudin-5 in cultured HBMEC after HG-IL1β insult with/without RGFP966 treatment. **b**–**e**. Quantifications of junction protein ZO-1, VE-cadherin, Occludin, and Claudin-5 protein expression in cultured HBMEC. Data are expressed as mean ± SEM. *P < 0.05 vs. norm, ^#^*P* < 0.05 vs. HG-IL1β, *n* = 6 cultures per groups. **f** Immunocytochemistry of ZO-1, VE-cadherin, and Claudin-5 in cultured HBMEC after HG-IL1β with/without RGFP966 treatment. White arrows indicate disrupted alignment of tight junction proteins

Next, we isolated the plasma membrane fractions of HBMEC and examined the junction protein expression by Western blot. NaK-ATPase served as an equal loading control of the membrane protein. In the HG-IL1β group, ZO-1, VE-cadherin, and Claudin-5 protein levels were significantly reduced by HG-IL1β insult, whereas this reduction was rescued by HDAC3 inhibition (Fig. 4a–e). We further performed immunocytochemistry to test the membrane localization of ZO-1, VE-cadherin, and Claudin-5 in cultured HBMECs. HG-IL1β insult may disrupt the alignment of tight junction proteins, while this disruption was rescued by HDAC3 inhibition (Fig. 4f).

### HDAC3 inhibition decreases Nrf2 expression and Keap1-Nrf2 interaction in db/db mice and in cultured HBMEC monolayer

**Fig. 5:**
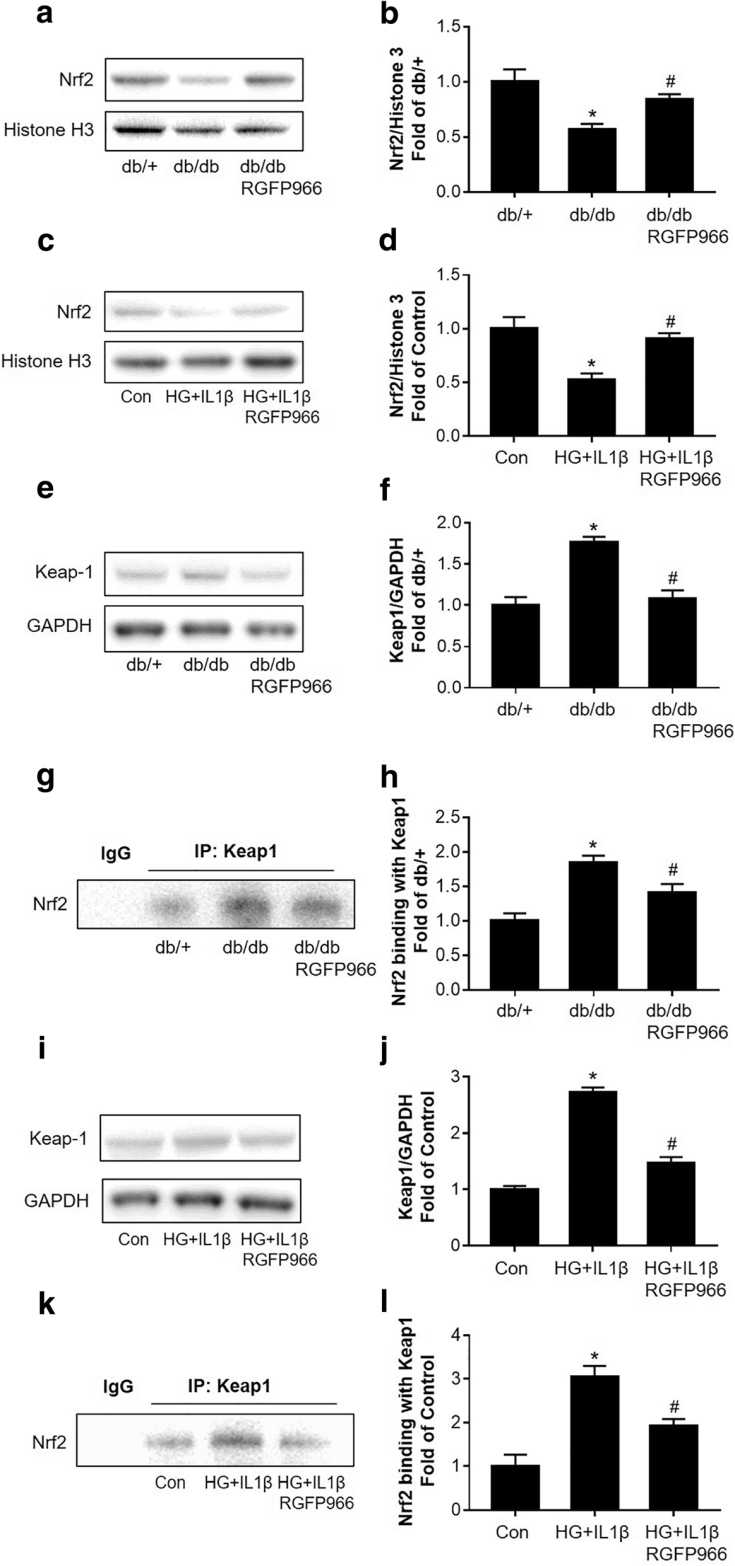
HDAC3 inhibition decreases Nrf2 expression and Keap1-Nrf2 interaction in db/db mice and in cultured HBMEC monolayer. **a**, **b** Representative Western blot gel images and quantification of Nrf2 protein expression in nuclear extract of the db/db and db/+ mouse brains. Histone H3 served as an equal loading control. Data are expressed as mean ± SEM.*P < 0.05 vs. db/+, ^#^*P* < 0.05 vs. db/db, *n* = 4 mice per group. **c**, **d** Representative Western blot gel image and quantification of Nrf2 protein expression in nuclear extract of HBMEC cultures. Histone H3 served as an equal loading control. Data are expressed as mean ± SEM. *P < 0.05 vs. norm, ^#^*P* < 0.05 vs. HG-IL1β, *n* = 4 cultures per group. **e**, **f** Representative Western blot gel images and quantification for Keap1 protein expression in the mouse brain. GAPDH served as an equal loading control. Data are expressed as mean ± SEM. *P < 0.05 vs. db/+, ^#^*P* < 0.05 vs. db/db, *n* = 4 mice per group. **g**, **h** Representative Western blot images and quantification for Nrf2 level (binding with keap-1) after Co-IP with Keap1 antibody using the brain lysates. Data are expressed as mean ± SEM. *P < 0.05 vs. db/+, ^#^*P* < 0.05 vs. db/db, *n* = 4 mice per group. **i**, **j** Representative Western blot gel images and quantification for Keap1 protein expression in cultured HBMEC. GAPDH served as an equal loading control. **k**, **l** Representative Western blot gel images and quantification for Nrf2 level (binding with keap-1) after Co-IP with Keap1 antibody in cultured HBMEC. Data are expressed as mean ± SEM. *P < 0.05 vs. norm, ^#^*P* < 0.05 vs. HG-IL1β, *n* = 4 cultures per group

We examined the Nrf2 protein expression levels of nuclear extraction by Western blots. We found that Nrf2 level was decreased in the db/db mouse brains compared to db/+ controls, while this decrease was significantly rescued by HDAC3 inhibition (Fig. 5a, b). Moreover, we found HG-IL1β insult significantly decreased Nrf2 protein level in nuclear extraction of cultured HBMEC, and HDAC3 inhibition significantly rescued the Nrf2 expression decrease (Fig. 5c, d). We also examined Nrf2’s repressor Keap1 protein expression and interaction with Nrf2. We found Keap1 protein expression was significantly increased in the db/db mouse brains compared to db/+ controls, while this increase was significantly attenuated by HDAC3 inhibition (Fig. 5e, f). We further performed Co-IP to test Keap1-Nrf2 interaction using Keap1 antibody, followed by Western blot using Nrf2 antibody. We observed significantly strengthened interaction of Keap1 with Nrf2 in the db/db mouse brain compared to db/+ controls, whereas this increase was significantly blocked by HDAC3 inhibition (Fig. 5g, h). For in vitro study, we further examined the Keap1 protein level in cultured HBMECs by Western blot. Consistent with the in vivo findings, Keap1 protein level in HBMECs cell lysate was significantly increased after HG-IL1β insult, which was significantly blocked by HDAC3 inhibition (Fig. 5i, j). Moreover, HDAC3 inhibition significantly reduced the interaction between Nrf2 and Keap1 of HG-IL1β insulted HBMEC cultures (Fig. 5k, l).

### HDAC3 inhibition affects miR-200a/Keap1/Nrf2 pathway and associated protective effects in endothelial monolayer permeability

**Fig. 6:**
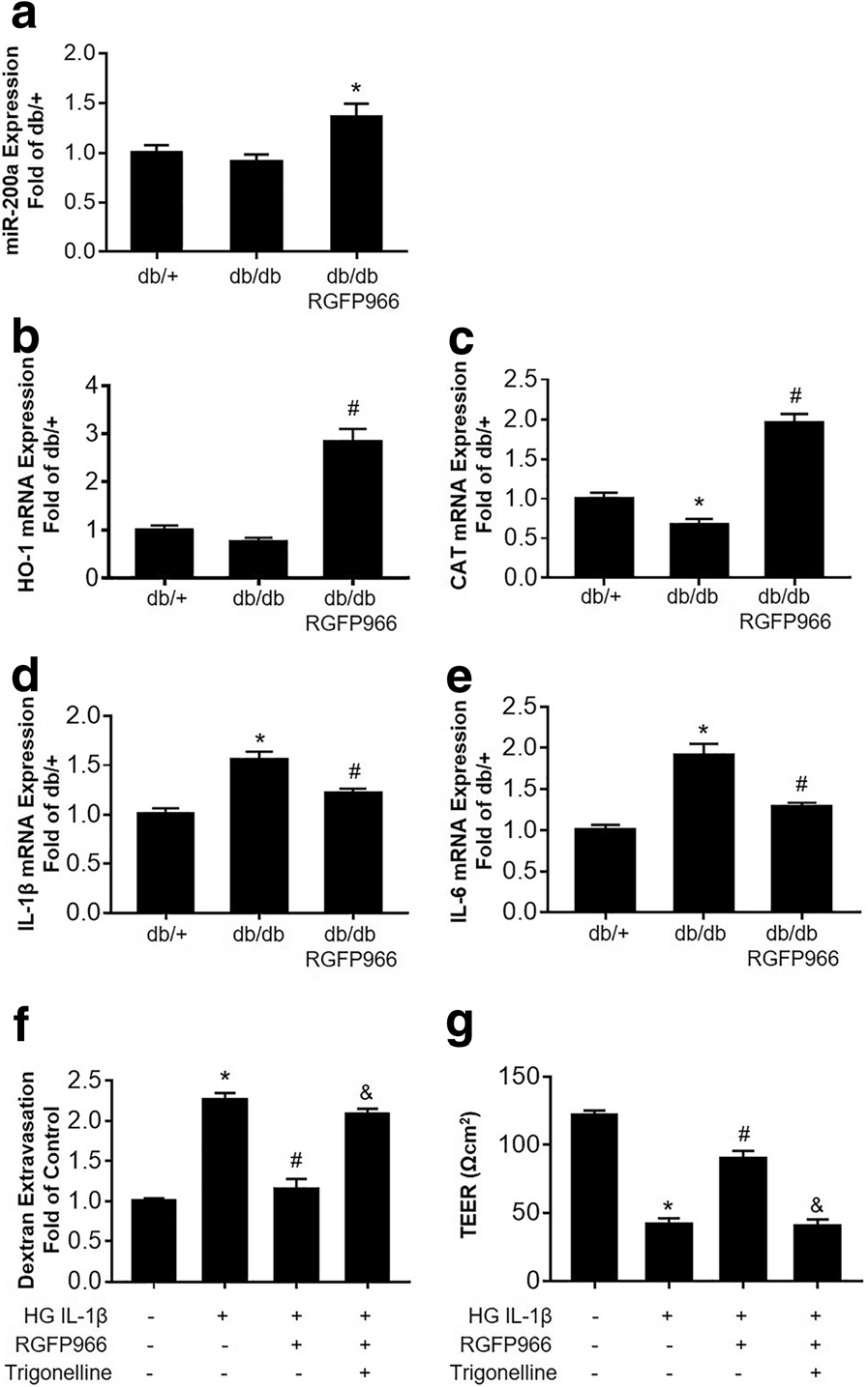
HDAC3 inhibition affects the miR-200a/Keap1/Nrf2 pathway and associated protective effects in endothelial monolayer permeability. **a** Quantification of miR-200a expression in db/+ and db/db mice with/without RGFP966 treatment, measured by real-time quantitative PCR. **b**–**e** Quantifications of HO-1, CAT, IL-1β, and IL-6 mRNA expression in db/+ and db/db mice with or without RGFP966 treatment, measured by real-time quantitative PCR. Data are expressed as mean ± SEM. *P < 0.05 vs. db/+, ^#^*P* < 0.05 vs. db/db, *n* = 4 mice per group. **f** Relative transendothelial permeability (Dextran Extravasation) of cultured HBMEC, induced by HG-IL1β insult and treated with RGFP966 or Nrf2 inhibitor trigonelline. **g** TEER measurement of HBMEC monolayers induced by HG-IL1β insult and treated with RGFP966 or Nrf2 inhibitor trigonelline. Data are expressed as mean ± SEM. *P < 0.05 vs. norm, ^#^*P* < 0.05 vs. HG-IL1β and *P* < 0.05 vs. HG-IL1β+RGFP966, *n* = 4 cultures per group

We then examined the expression of miR-200a under HDAC3 inhibition. We found that miR-200a levels were not significantly different in the mouse brains between db/db and db/+ mice. However, treatment with the RGFP966 leads to a significant increase in miR-200a levels (Fig. 6a). To verify the involvement of the Nrf2 in the protective role of HDAC3 inhibition in BBB permeability, we examined the expression of Nrf2 targeting (upregulating) genes Catalase (CAT) and Heme oxygenase-1 (HO-1). HDAC3 inhibition significantly increased the mRNA levels of CAT and HO-1, compared to the vehicle-treated db/db mice (Fig. 6b, c). Furthermore, the mRNA levels of Nrf2-supressing genes and pro-inflammatory cytokines such as IL-1β and IL-6 were decreased after HDAC3 inhibition (Fig. 6d, e). These findings indicate that Nrf2 signaling was activated by HDAC3 inhibition. We further validated whether Nrf2 is required for the protection effect of HDAC3 inhibition on HG-IL1β-induced endothelial monolayer permeability. HBMEC monolayer with HG-IL1β insult was treated with RGFP966 alone or RGFP966 combined with Nrf2 inhibitor trigonelline (1 μM). We found that trigonelline significantly blocked the protection effect of HDAC3 inhibition in HG-IL1β-induced endothelial monolayer permeability (Fig. 6f, g). These data suggesting that miR-200a/Keap1/Nrf2 is required for HDAC3 protection of BBB integrity.

## Discussion

Accumulating in vivo and in vitro studies have demonstrated that T2DM can progressively compromise BBB integrity and may significantly contribute T2DM-associated neurological disorders. For example, significant extravasation of plasma proteins (e.g., 60 kDa albumin) has been reported in diabetic rodents [27] and humans [28]. It is therefore of high clinical importance to clarify the mechanisms of T2DM-induced BBB dysfunction. In this study, we for the first time investigated the effect of HDAC3 inhibition on BBB permeability using both diabetic mice and cultured HBMEC monolayer. We found that (1) the expression and activity of HDAC3 are increased in db/db mice in the hippocampus and cortex, (2) HDAC3 inhibition ameliorated BBB permeability in db/db mice and HBMEC monolayer permeability after HG-IL1β insult, (3) HDAC3 inhibition rescued junction proteins loss induced by diabetic state, (4) the protection effect of HDAC3 inhibition on BBB permeability relies on Nrf2 activation, (5) HDAC3 inhibition upregulates Nrf2 by decreasing the binding of Nrf2 to keap1, the negative regulator of Nrf2, which is associated with miR-200a upregulation.

HDACs comprise a family of 18 enzymes that are referred to as HDAC 1–11 and SIRT 1–7. They are classified into four groups based on function and sequence homology: class I (HDACs1–3 and 8), class II (HDACs 4–7, 9–10), class III (SIRT1–7), and class IV (HDAC11) [29]. HDAC3 is the most highly expressed class I HDAC in the brain [9]. By using HDAC3 knockout mice and highly selective HDAC3 inhibitor, increasing evidence support that HDAC3 is involved in the pathophysiology of diabetes mellitus and many neurological disorders [11, 12, 30, 31]. Consistently, in this study, we observed an increase of HDAC3 activity and expression in the hippocampus and cortex of db/db mice. We previously found that HDAC3 inhibition can prevent OGD/R-induced transendothelial permeability in cultured HBMEC [19]. In this current study, we found that HDAC3 inhibition by RGFP966 inhibited the activity of HDAC3 and reduced BBB permeability in db/db mice and HBMEC monolayer permeability after HG-IL1β insult. These findings altogether indicate that HDAC3 may play critical roles in T2DM-associated neuronal disorders and could be a potential target for therapeutics development.

We found BBB permeability of NaFl was significantly increased in db/db compared to db/+ mice. Previous investigations suggest the changes in BBB permeability to NaFl may be the earliest and the most sensitive indicator of BBB disruption than other tracers [32]. The increased NaFl permeability is consistent with previous reports that there is an early BBB damage at this age (~ 16 weeks) of db/db mice [21, 26].

Junction proteins, including tight junction and adherens junction that are located in the apical region of the cell membrane, are critical components and regulators of BBB permeability [33]. Various pathological conditions such as ischemia/hypoxia and neuroinflammation may lead to disruption of BBB integrity via loss of junction proteins, which results in entrance of numerous activated pro-inflammatory cells and protein-rich influx into the brain parenchyma, enhancing overall cerebral pathology [34]. In this study, we examined the junction protein expression in a diabetic state. We found HDAC3 inhibition significantly rescued downregulation of junction proteins VE-Cadherin and Claudin-5 but not ZO-1 and Occludin. HDAC3 inhibition rescued diabetes-induced junction proteins loss, which might contribute to the protective effect of HDAC3 inhibition on diabetic state-induced BBB permeability. It has been well known that tight junction expressions are controlled with a variety of regulatory mechanisms including Nrf2 [35]. However, emerging experimental investigations showed different regulation roles or effects by different HDAC family members or HDAC inhibitors, and these effects might also vary under different pathological conditions in modulating different junction proteins [36]. This may explain that, in our study, HDAC3 inhibition only have protective effects on some junction proteins only.

Recent studies using Nrf2 activators demonstrated that Nrf2 protects against BBB dysfunction in multiple neurological disorders [37, 38]. Under basal conditions, Nrf2 is sequestered in the cytoplasm by a repressor protein Keap1, which is a repressor protein that binds to Nrf2 and promotes its degradation by the ubiquitin-proteasome pathway [39]. Reduced Keap1 expression can lead to nuclear translocation of Nrf2 and subsequent target gene expression. This Nrf2-Keap1 signaling pathway has been well described as the major mechanism involved in cellular defense against oxidative stress in many pathological conditions, including T2DM [40].

Our group has proved that recombinant FGF21 protects against BBB leakage through Nrf2 upregulation in type 2 diabetes mice [21]. In this study, we found that HDAC3 inhibition may protect against diabetes-induced BBB damage through Nrf2 activation*.* HDAC3 inhibition lead to the decrease of Keap1 protein, a repressor of Nrf2. Nrf2 then translocate into the nucleus and promote the transcription of protective gene CAT and HO-1. This finding is consistent with previous studies that Nrf2-dependent antioxidant genes were upregulated in HDAC3-deficient macrophages [41]. We also found that HDAC3 inhibition effectively reduced the mRNA levels of IL-1β and IL-6 in db/db mice, which is consistent with the facts that upregulation of Nrf2 downstream proteins such as HO-1 can reduce the production of pro-inflammatory cytokines [42] and that HDAC3 inhibition may reduce pro-inflammatory response by cytokine downregulation [43].

It has been reported that HDAC3 inhibition regulates Keap1/Nrf2 in tumor cell lines or type 1 diabetes-associated aortic pathologies, which is through modulating the expression of miR-200a that binds to the 3′-terminal region of the Keap1 mRNA to inhibit its translation [17, 18]. In this study, we observed that treatment with the RGFP966 leads to a significant increase in miR-200a levels, which may one of the underlying mechanisms for the Keap1 downregulation and associated Nrf2 activation.

Since BBB leakage in type 2 diabetes model was reported mainly located in the brain hippocampus [24] and cortex [25], the hippocampus and cortex areas are focused on this study. Unfortunately, a technical difficulty is that tissue or nuclear protein amount of isolated cerebral microvascular fragments in mice are too limited to assess HDAC3 and Nrf2 activity or activation. We therefore not only, in this study, assessed HDAC3 mRNA, protein, and activity using brain hippocampus and cortex tissues, but also tested our hypothesis in cultured HBMEC under in vitro diabetes condition and hyperglycemia plus IL-1β exposure.

Besides, there are a few caveats in this study. First, the C57BLKS-Leprdb T2DM mice that are deficient in leptin receptor may not reflect the actual pathology in T2DM patients. There are variable mechanisms and metabolic characteristics among different T2DM animal models; thus, studies using other diabetes models should be considered in the future. Second, since Nrf2-keap 1 pathway signaling also involves in astrocytes [44], another important BBB cellular component [45, 46], astrocytes, might also play important roles. However, we did not use co-culture of endothelial cells with primary astrocytes (in vitro BBB model) [47], and there was also a lack of astrocyte-conditioned media in the in vitro portion of the present study. The detailed roles and mechanisms of astrocytes in HDAC3-mediated BBB permeability of T2DM warrant investigation in the future. Third, it has been known that the function of HDAC3 consists of as part of nuclear receptor co-repressors, its enzymatic activity and its post-translational modifications [48]. Thus, the pathological role of HDAC3 in the T2DM-associated BBB permeability is likely complex, which requires further investigation. Fourth, there was a discrepancy in assessing transendothelial permeability by tracing 70 kDa FITC-Dextran in the cultured monolayer endothelial cell cultures, but we only found BBB leakage increase of small molecule NaFl, but not 4–10 kDa FITC-Dextran in the db/db mouse brain. Because BBB integrity in the brain tissue is much tighter than the monolayer endothelial cell culture, transendothelial permeability was commonly assessed by tracing relatively larger molecules in monolayer endothelial cell cultures [49]. Using a more physically comprehensive in vitro BBB model would help to elucidate more detailed underlying mechanisms of HDAC3 inhibition-associated BBB protection in the future [50]. Fifth, we observed a coincidence HDAC3 inhibition-associated junction protein expression promotion and BBB permeability protection, but their causal relation remains to be defined. Last, it has been well known that BBB leakage in T2DM may lead to cognitive impairment [26, 51], which was not examined in the present study; however, the role and mechanism of HDAC3 inhibition in the BBB-associated neurological complication such as cognitive impairment warrant further investigation.

## Conclusion

Experimental findings from the present study show that HDAC3 inhibition may protect against T2DM-induced BBB permeability, and the beneficial effect is at least in part attributed by miR-200a/Keap1/Nrf2 pathway. Taken together, this experimental study suggests that HDAC3 might be a new therapeutic target for BBB damage in T2DM.

## Additional files

Additional file 1: Animal numbers used in each experimental assessment. Totally, four groups of mice were used in each experiment. T2DM (db/db, leptin receptor-deficient), genetic non-hyperglycemic control (db/+), and wild-type (WT) male mice at the age of 16 weeks were used in this study. One group of db/db mice were treated with HDAC3 inhibitor RGFP966 (db/db+RGFP966). In each experiment, animal numbers used for each group were listed. (DOCX 14 kb)

Additional file 2: Changes of animal body weight and blood glucose level. Sixteen-week-old db/db male mice were treated with/without RGFP966 for 10 days. Body weight (a) and blood glucose level (b) were measured every other day after RGFP966. There was no statistic difference between RGFP966 treated and non-treated db/db mice. Data are expressed as mean ± SEM. *n* = 8 mice per group. (TIF 545 kb)

Additional file 3: Baseline levels of HDAC3 expression and activity in the brain of C57BLKS/J and db/+ mice. a. Representative Western blot gel images for HDAC3 in the same genetic background WT mice-C57BLKS/J (BKS) mice and db/+ mice. b. Quantification of Western blot analysis of HDAC3 protein levels. GAPDH served as an equal loading control. c. Quantification of HDAC3 mRNA expression examined by real-time quantitative PCR. d. Quantification of HDAC3 activity in nuclear extraction by HDAC3 activity assay. Data are expressed as mean ± SEM. *n* = 6 mice per group. (TIF 297 kb)

## Abbreviations

BBB: Blood-brain barrier
CAT: Catalase
CNS: Central nervous system
Co-IP: Co-immunoprecipitation
DM: Diabetes mellitus
FITC: Fluorescein isothiocyanate
HAT: Histone acetyltransferase
HBMEC: Human brain microvascular endothelial cell
HDAC3: Histone deacetylase 3
HO-1: Heme oxygenase-1
IL-1β: Interleukin 1 beta
NaFl: Sodium fluorescein
Nrf2: Nuclear factor-E2-related factor 2
OGD/R: Oxygen-glucose deprivation/reoxygenation
PCR: Real-time polymerase chain reaction
T2DM: Type 2 diabetes mellitus
TEER: Trans-endothelial electrical resistance

## References

[1] Zaccardi F, Webb DR, Yates T, Davies MJ (2016). Pathophysiology of type 1 and type 2 diabetes mellitus: a 90-year perspective. Postgrad Med J.

[2] Roney C, Kulkarni P, Arora V, Antich P, Bonte F, Wu A (2005). Targeted nanoparticles for drug delivery through the blood-brain barrier for Alzheimer’s disease. J Control Release.

[3] Weiss N, Miller F, Cazaubon S, Couraud PO (2009). The blood-brain barrier in brain homeostasis and neurological diseases. Biochim Biophys Acta.

[4] Shao B, Bayraktutan U (2013). Hyperglycaemia promotes cerebral barrier dysfunction through activation of protein kinase C-beta. Diabetes Obes Metab.

[5] Zhao F, Deng J, Xu X, Cao F, Lu K, Li D (2018). Aquaporin-4 deletion ameliorates hypoglycemia-induced BBB permeability by inhibiting inflammatory responses. J Neuroinflammation.

[6] Hawkins BT, Davis TP (2005). The blood-brain barrier/neurovascular unit in health and disease. Pharmacol Rev.

[7] Strahl BD, Allis CD (2000). The language of covalent histone modifications. Nature..

[8] Malvaez M, McQuown SC, Rogge GA, Astarabadi M, Jacques V, Carreiro S (2013). HDAC3-selective inhibitor enhances extinction of cocaine-seeking behavior in a persistent manner. Proc Natl Acad Sci U S A.

[9] Broide RS, Redwine JM, Aftahi N, Young W, Bloom FE, Winrow CJ (2007). Distribution of histone deacetylases 1-11 in the rat brain. J Mol Neurosci.

[10] Chou DH, Holson EB, Wagner FF, Tang AJ, Maglathlin RL, Lewis TA (2012). Inhibition of histone deacetylase 3 protects beta cells from cytokine-induced apoptosis. Chem Biol.

[11] Lundh M, Galbo T, Poulsen SS, Mandrup-Poulsen T (2015). Histone deacetylase 3 inhibition improves glycaemia and insulin secretion in obese diabetic rats. Diabetes Obes Metab.

[12] Sathishkumar C, Prabu P, Balakumar M, Lenin R, Prabhu D, Anjana RM (2016). Augmentation of histone deacetylase 3 (HDAC3) epigenetic signature at the interface of proinflammation and insulin resistance in patients with type 2 diabetes. Clin Epigenetics.

[13] Sathishkumar C, Prabu P, Mohan V, Balasubramanyam M (2018). Linking a role of lncRNAs (long non-coding RNAs) with insulin resistance, accelerated senescence, and inflammation in patients with type 2 diabetes. Hum Genomics.

[14] Dinkova-Kostova AT, Abramov AY (2015). The emerging role of Nrf2 in mitochondrial function. Free Radic Biol Med.

[15] Alfieri A, Srivastava S, Siow RC, Modo M, Fraser PA, Mann GE (2011). Targeting the Nrf2-Keap1 antioxidant defence pathway for neurovascular protection in stroke. J Physiol.

[16] Zhao J, Moore AN, Redell JB, Dash PK (2007). Enhancing expression of Nrf2-driven genes protects the blood-brain barrier after brain injury. J Neurosci.

[17] Murray-Stewart T, Hanigan CL, Woster PM, Marton LJ, Casero RA (2013). Histone deacetylase inhibition overcomes drug resistance through a miRNA-dependent mechanism. Mol Cancer Ther.

[18] Zhang J, Xu Z, Gu J, Jiang S, Liu Q, Zheng Y (2018). HDAC3 inhibition in diabetic mice may activate Nrf2 preventing diabetes-induced liver damage and FGF21 synthesis and secretion leading to aortic protection. Am J Physiol Endocrinol Metab.

[19] Zhao Q, Yu Z, Zhang F, Huang L, Xing C, Liu N, et al. HDAC3 inhibition prevents oxygen glucose deprivation/reoxygenation-induced transendothelial permeability by elevating PPARgamma activity in vitro. J Neurochem. 2018. 10.1111/jnc.14619 30347434

[20] Sajja RK, Prasad S, Tang S, Kaisar MA, Cucullo L (2017). Blood-brain barrier disruption in diabetic mice is linked to Nrf2 signaling deficits: role of ABCB10?. Neurosci Lett.

[21] Yu Zhanyang, Lin Li, Jiang Yinghua, Chin Ian, Wang Xiaojie, Li Xiaokun, Lo Eng H., Wang Xiaoying (2018). Recombinant FGF21 Protects Against Blood-Brain Barrier Leakage Through Nrf2 Upregulation in Type 2 Diabetes Mice. Molecular Neurobiology.

[22] Guo S, Lok J, Zhao S, Leung W, Som AT, Hayakawa K (2016). Effects of controlled cortical impact on the mouse brain vasculome. J Neurotrauma.

[23] Lin L, Wang Q, Qian K, Cao Z, Xiao J, Wang X (2018). bFGF protects against oxygen glucose deprivation/reoxygenation-induced endothelial monolayer permeability via S1PR1-dependent mechanisms. Mol Neurobiol.

[24] Yoo DY, Yim HS, Jung HY, Nam SM, Kim JW, Choi JH (2016). Chronic type 2 diabetes reduces the integrity of the blood-brain barrier by reducing tight junction proteins in the hippocampus. J Vet Med Sci.

[25] Min LJ, Mogi M, Shudou M, Jing F, Tsukuda K, Ohshima K (2012). Peroxisome proliferator-activated receptor-gamma activation with angiotensin II type 1 receptor blockade is pivotal for the prevention of blood-brain barrier impairment and cognitive decline in type 2 diabetic mice. Hypertension.

[26] Stranahan AM, Hao S, Dey A, Yu X, Baban B (2016). Blood-brain barrier breakdown promotes macrophage infiltration and cognitive impairment in leptin receptor-deficient mice. J Cereb Blood Flow Metab.

[27] Fujihara R, Chiba Y, Nakagawa T, Nishi N, Murakami R, Matsumoto K (2016). Albumin microvascular leakage in brains with diabetes mellitus. Microsc Res Tech.

[28] van Harten AC, Jongbloed W, Teunissen CE, Scheltens P, Veerhuis R, van der Flier WM (2017). CSF ApoE predicts clinical progression in nondemented APOEepsilon4 carriers. Neurobiol Aging.

[29] de Ruijter AJ, van Gennip AH, Caron HN, Kemp S, van Kuilenburg AB (2003). Histone deacetylases (HDACs): characterization of the classical HDAC family. Biochem J.

[30] Yang X, Wu Q, Zhang L, Feng L (2016). Inhibition of histone deacetylase 3 (HDAC3) mediates ischemic preconditioning and protects cortical neurons against ischemia in rats. Front Mol Neurosci.

[31] Park MJ, Sohrabji F (2016). The histone deacetylase inhibitor, sodium butyrate, exhibits neuroprotective effects for ischemic stroke in middle-aged female rats. J Neuroinflammation.

[32] Kaya M, Ahishali B (2011). Assessment of permeability in barrier type of endothelium in brain using tracers: Evans blue, sodium fluorescein, and horseradish peroxidase. Methods Mol Biol.

[33] Paris L, Tonutti L, Vannini C, Bazzoni G (2008). Structural organization of the tight junctions. Biochim Biophys Acta.

[34] Na W, Shin JY, Lee JY, Jeong S, Kim WS, Yune TY (2017). Dexamethasone suppresses JMJD3 gene activation via a putative negative glucocorticoid response element and maintains integrity of tight junctions in brain microvascular endothelial cells. J Cereb Blood Flow Metab.

[35] Harhaj NS, Antonetti DA (2004). Regulation of tight junctions and loss of barrier function in pathophysiology. Int J Biochem Cell Biol.

[36] Bordin M, D'Atri F, Guillemot L, Citi S (2004). Histone deacetylase inhibitors up-regulate the expression of tight junction proteins. Mol Cancer Res.

[37] Sajja RK, Green KN, Cucullo L (2015). Altered Nrf2 signaling mediates hypoglycemia-induced blood-brain barrier endothelial dysfunction in vitro. PLoS One.

[38] Zeng J, Chen Y, Ding R, Feng L, Fu Z, Yang S (2017). Isoliquiritigenin alleviates early brain injury after experimental intracerebral hemorrhage via suppressing ROS- and/or NF-kappaB-mediated NLRP3 inflammasome activation by promoting Nrf2 antioxidant pathway. J Neuroinflammation.

[39] Taguchi K, Motohashi H, Yamamoto M (2011). Molecular mechanisms of the Keap1-Nrf2 pathway in stress response and cancer evolution. Genes Cells.

[40] David JA, Rifkin WJ, Rabbani PS, Ceradini DJ (2017). The Nrf2/Keap1/ARE pathway and oxidative stress as a therapeutic target in type II diabetes mellitus. J Diabetes Res.

[41] Chen X, Barozzi I, Termanini A, Prosperini E, Recchiuti A, Dalli J (2012). Requirement for the histone deacetylase Hdac3 for the inflammatory gene expression program in macrophages. Proc Natl Acad Sci U S A.

[42] Clerigues V, Guillen MI, Castejon MA, Gomar F, Mirabet V, Alcaraz MJ (2012). Heme oxygenase-1 mediates protective effects on inflammatory, catabolic and senescence responses induced by interleukin-1beta in osteoarthritic osteoblasts. Biochem Pharmacol.

[43] Xia M, Zhao Q, Zhang H, Chen Y, Yuan Z, Xu Y (2017). Proteomic analysis of HDAC3 selective inhibitor in the regulation of inflammatory response of primary microglia. Neural Plast.

[44] Baxter PS, Hardingham GE (2016). Adaptive regulation of the brain’s antioxidant defences by neurons and astrocytes. Free Radic Biol Med.

[45] Nag S (2011). Morphology and properties of astrocytes. Methods Mol Biol.

[46] Zhao Z, Nelson AR, Betsholtz C, Zlokovic BV (2015). Establishment and dysfunction of the blood-brain barrier. Cell.

[47] He Y, Yao Y, Tsirka SE, Cao Y (2014). Cell-culture models of the blood-brain barrier. Stroke.

[48] Emmett MJ, Lazar MA. Integrative regulation of physiology by histone deacetylase 3. Nat Rev Mol Cell Biol. 2018. 10.1038/s41580-018-0076-0 PMC6347506 30390028

[49] Helms HC, Abbott NJ, Burek M, Cecchelli R, Couraud PO, Deli MA (2016). In vitro models of the blood-brain barrier: an overview of commonly used brain endothelial cell culture models and guidelines for their use. J Cereb Blood Flow Metab.

[50] Wilhelm I, Krizbai IA (2014). In vitro models of the blood-brain barrier for the study of drug delivery to the brain. Mol Pharm.

[51] Bogush M, Heldt NA, Persidsky Y (2017). Blood brain barrier injury in diabetes: unrecognized effects on brain and cognition. J Neuroimmune Pharmacol..

