# Supplementary material for: HDAC3 inhibition prevents blood-brain barrier permeability through Nrf2 activation in type 2 diabetes male mice
Source: J Neuroinflammation. 2019 May 17;16:103. doi: 10.1186/s12974-019-1495-3 (PMC6525453; doi:10.1186/s12974-019-1495-3)
Supplement: Supplementary file 1 — Animal numbers used in each experimental assessment. Totally, four groups of mice were used in each experiment. T2DM (db/db, leptin receptor-deficient), genetic non-hyperglycemic control (db/+), and wild-type (WT) male mice at the age of 16 weeks were used in this study. One group of db/db mice were treated with HDAC3 inhibitor RGFP966 (db/db+RGFP966). In each experiment, animal numbers used for each group were listed. (DOCX 14 kb) [file 12974_2019_1495_MOESM1_ESM.docx]

| Animal numbers uses in each experimental assessment | | | | |
| --- | --- | --- | --- | --- |
| Groups | WT | db+ | db/db | db/db + RGFP966 |
| HDAC3 protein expression | 6 | 6 | 6 | 6 |
| HDAC3 activity | 6 | 6 | 6 | 6 |
| HDAC3 mRNA expression | 6 | 6 | 6 | 6 |
| Fluorescent image |  | 5 | 5 | 5 |
| NaFI leakage |  | 5 | 5 | 5 |
| Tight junction expression |  | 5 | 5 | 5 |
| miRNA200a /Nrf2/ Keap 1 expression |  | 8 | 8 | 8 |
| Nrf2 IP |  | 4 | 4 | 4 |
| Total | 18 | 45 | 45 | 45 |
