# Supplementary figures and images for: HDAC3 inhibition prevents blood-brain barrier permeability through Nrf2 activation in type 2 diabetes male mice
Source: J Neuroinflammation. 2019 May 17;16:103. doi: 10.1186/s12974-019-1495-3 (PMC6525453; doi:10.1186/s12974-019-1495-3)

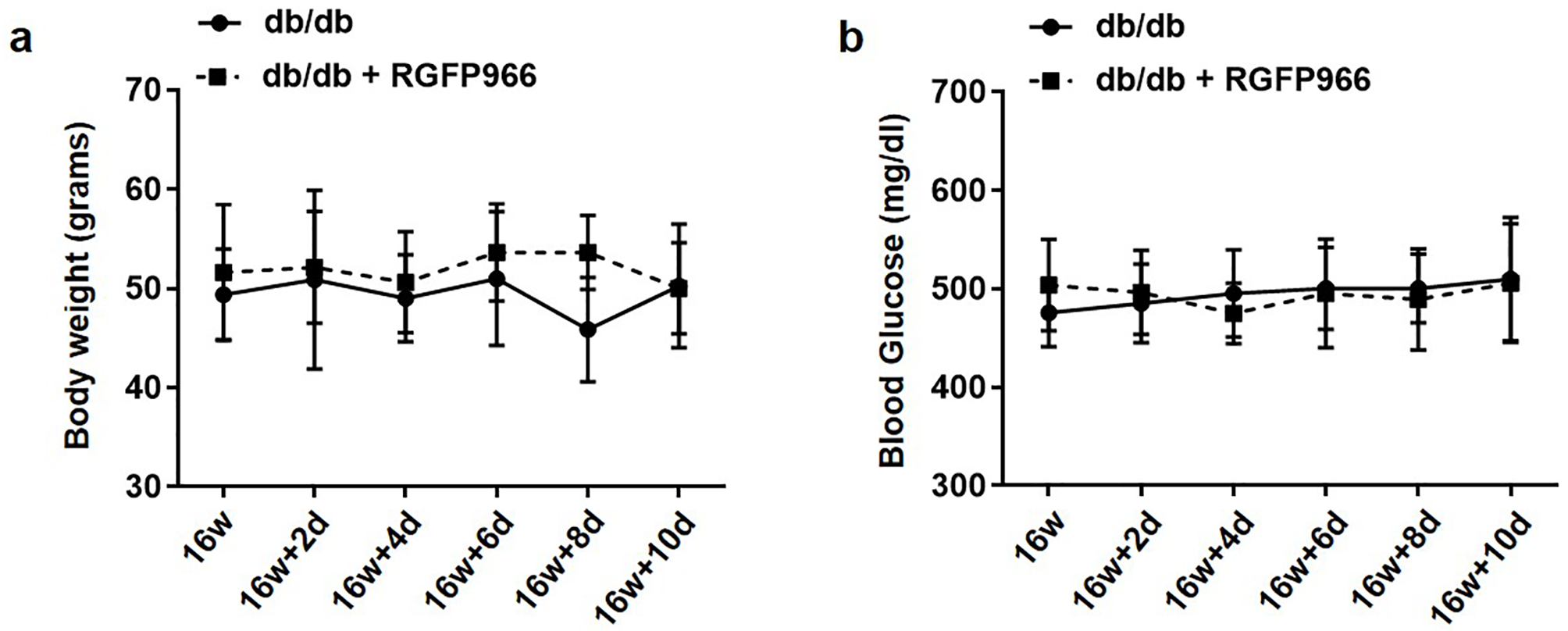

Supplement: Supplementary file 2 — Changes of animal body weight and blood glucose level. Sixteen-week-old db/db male mice were treated with/without RGFP966 for 10 days. Body weight (a) and blood glucose level (b) were measured every other day after RGFP966. There was no statistic difference between RGFP966 treated and non-treated db/db mice. Data are expressed as mean ± SEM. n = 8 mice per group. (TIF 545 kb) [file 12974_2019_1495_MOESM2_ESM.tif]

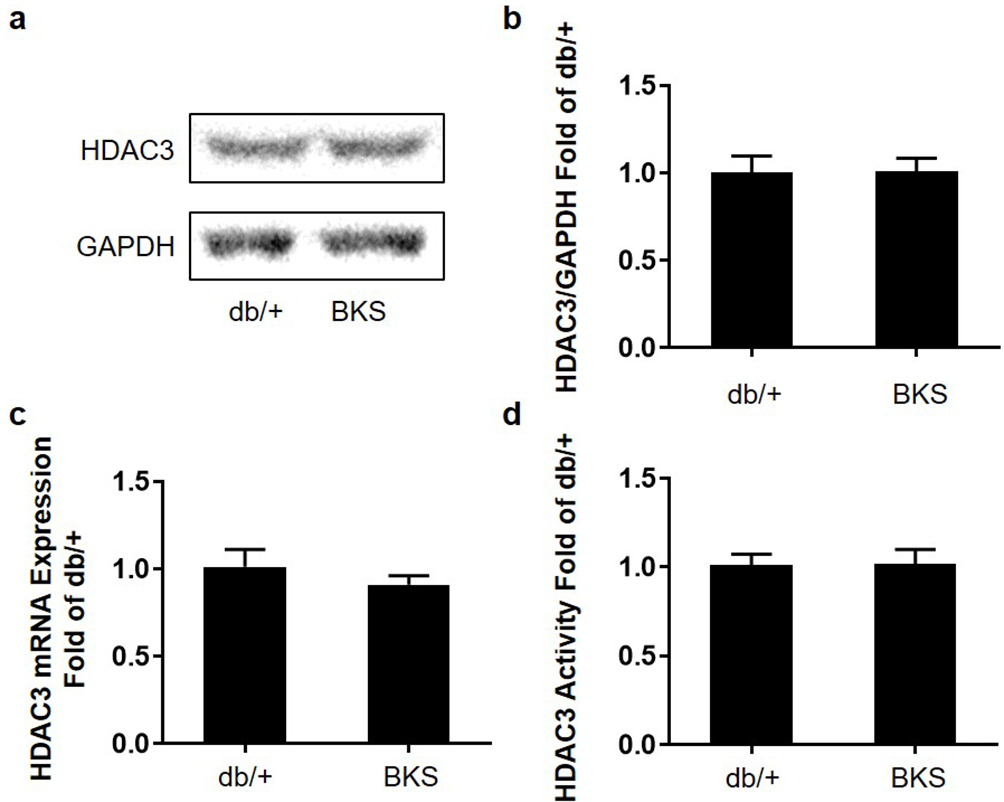

Supplement: Supplementary file 3 — Baseline levels of HDAC3 expression and activity in the brain of C57BLKS/J and db/+ mice. a. Representative Western blot gel images for HDAC3 in the same genetic background WT mice-C57BLKS/J (BKS) mice and db/+ mice. b. Quantification of Western blot analysis of HDAC3 protein levels. GAPDH served as an equal loading control. c. Quantification of HDAC3 mRNA expression examined by real-time quantitative PCR. d. Quantification of HDAC3 activity in nuclear extraction by HDAC3 activity assay. Data are expressed as mean ± SEM. n = 6 mice per group. (TIF 297 kb) [file 12974_2019_1495_MOESM3_ESM.tif]
